# Supplementary material for: Expanding the genetic toolkit of Tribolium castaneum
Source: PLoS One. 2018 Apr 12;13(4):e0195977. doi: 10.1371/journal.pone.0195977 (PMC5897005; doi:10.1371/journal.pone.0195977)
Supplement: S1 Table — (DOCX) [file pone.0195977.s001.docx]

| **Primer** | **Sequence (5’ 🡪 3’)** | **Application** |
| --- | --- | --- |
| pTC006550-F | GATCGATATCGGCGCGCCAACAGC | Amplification of the *TC006550* promoter from blaAmp-Tc6550Pro-GFPZeo-Luciferase-HSP-Orange-pIZT |
| pTC006550-R | CTCTAGTACCAACCTTACGTTAGAATTGAGTTACGAG |  |
| p130der-F | ACGTAAGGTTGGTACTAGAGGTACACGTCTCCC | Linearization of p130der for insertion of the *TC006550* promoter |
| p130der-R | TTGGCGCGCCGATATCGATCGCGCGCAGA |  |
| pTC006550-GAL4-SV40-F | CATAGGCCACGGCGCGCCAACA | Amplification of the *ribo*-GAL4 coding sequence from p130der |
| pTC006550-GAL4-SV40-R | CGGAGTGGACAGATACATTGATGAGTTTGGACAAACCAC |  |
| pBac-F | CAATGTATCTGTCCACTCCGCCTTTAGTTTGATTATAATACA | Linearization of pBac[3xP3-EGFP] for insertion of the *ribo*-GAL4 coding sequence |
| pBac-R | TTGGCGCGCCGTGGCCTATGGCATTATTGTACGGA |  |
| nls-EGFP-F | ACTAGTGAATTCAAAGTACCACTCGAGAGCATGGCTCCAAAGAAAGAAGCGTAAGGTAAAT | Amplification of nls-EGFP from pSYC-102 |
| nls-EGFP-R | CCTTAAGCTTGTACAGCTCGTCCATGCCGA |  |
| T2A-mCherry-CAAX-F | TCGGCATGGACGAGCTGTACAAGCTTAAGG | Amplification of T2A-mCherry-CAAX from pSYC-102 |
| T2A-mCherry-CAAX-R | GTGGTATGGCTGATTATGATCTAGAGTCGCTCAGGAGAGCACACACTTGCAGCTCATGCA |  |
